# Ultrasound-assisted deep eutectic solvent-mediated nano-lignin isolation from *Prunus persica* endocarp for broad-spectrum sunscreen application

**DOI:** 10.1039/d5ra08798h

**Published:** 2026-04-10

**Authors:** Tsegaye Abera Mekuriaw, Belachew Zegale Tizazu, Adamu Esubalew Kassa, Yalew Woldeamanuel Sitotaw

**Affiliations:** a Department of Chemical Engineering, College of Engineering, Addis Ababa Science and Technology University P.O. Box 16417 Addis Ababa Ethiopia; b Biotechnology and Bioprocess Center of Excellence, Addis Ababa Science and Technology University P.O. Box 16417 Addis Ababa Ethiopia belachew.zegale@aastu.edu.et

## Abstract

Chemical sunscreens are widely used nowadays because of their capacity to protect the skin from ultraviolet radiation. However, commercial chemical sunscreens provide limited sun protection factor and broad-spectrum protection, typically ultraviolet A_1_ (UVA_1_), which covers wavelengths from 340 to 400 nm. Thus, in this study, ultrasound-assisted DES-mediated nano-lignin isolation was performed to investigate the potential of nano-lignin to improve the sun protection factor (SPF) value and broaden the UV absorption spectrum, thereby lowering transmittance in the wavelength range of 290–400 nm. As a result, nano-lignin was mixed with an SPF 27 commercial sunscreen (CSS), and the SPF value and broad-spectrum coverage were evaluated. The obtained results showed substantial improvements in the SPF value and broadened the UV absorption spectrum, which corresponded with the amount of nano-lignin incorporated. At 1 wt% of nano-lignin, the SPF value improved from control SPF 27 to SPF 30.65. Accordingly, increasing the amount of nano-lignin concentration to 5 wt% and 10 wt% increased the SPF values significantly to 36.75 and 48.59, respectively, under similar conditions. Correspondingly, the UV absorption spectra of the formulations were analyzed to evaluate the changes in the absorbance range relevant to UVA_1_, and a promising result was obtained with10 wt% of nano-lignin addition. The improvements in the SPF value and spectrum range could be attributed to the synergistic effects of the aromatic functional groups in nano-lignin and chemical sunscreen actives in CSS. Thus, nano-lignin can be considered a possible alternative to chemical sunscreen actives.

## Abstract 2

Chemical sunscreens are widely used nowadays because of their capacity to protect the skin from ultraviolet radiation.

## Graphical abstract

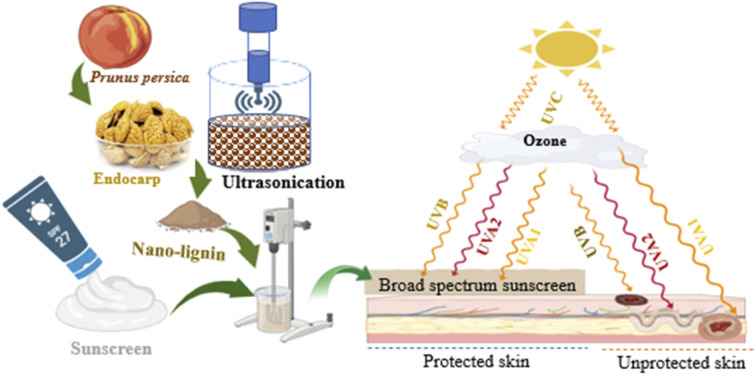

## 1 Introduction

Lignin is the second most abundant component of lignocellulosic biomass, containing aromatic structures with ultraviolet (UV)-absorbing and antioxidant properties. These properties of lignin make it a key element in the cosmetic industry and an active ingredient in sunscreen formulation. Sunscreen is a crucial product that is used every day to minimize the negative effects of extreme sunlight exposure, such as photodamage, photoaging, and skin cancer.^1^ To mitigate the effects of UV radiation, chemical and physical sunscreens are being used for a long time.^2^

Physical sunscreens typically contain titanium dioxide and zinc oxide, which protect the skin from ultraviolet B (UVB from 280–315 nm) and ultraviolet A (UVA from 315–400 nm) radiation, respectively, by scattering and reflecting UV radiation.^3^ Physical sunscreens are less comfortable on the skin due to their chalky white appearance.^4^ Thus, reducing the particle size or blending with herbal UV absorbers (active UV filters), including plant extracts such as oils, can make physical sunscreens more pleasant and comfortable. However, such UV absorbers have limitations due to their intensive extraction process and low UV absorption efficiency.^5^ Chemical sunscreens, which contain organic and inorganic filters, have been developed to overcome the limitations of physical sunscreens. They are widely used nowadays due to their efficiency in protecting the skin from harmful ultraviolet radiations by absorbing, scattering, and blocking UV radiation.^2^ However, despite these advantages, chemical sunscreens exhibit poor photostability and offer insufficient filtering of broad-spectrum UV radiations (UVA_1_, 340–400 nm).^6^ Thus, researchers nowadays are searching for broad-spectrum UV filters derived from organic polymers for enhancing photostability and sun protection efficiency and to alleviate the negative effects of broad-spectrum UV radiations at least within 370 nm, adhering to the US FDA standards.^6^

Recently, lignin was found to be a safe and ecofriendly UV-absorbing ingredient for sunscreens due to its aromatic and active functional groups, such as the methoxy, phenol, carbonyl, and hydroxyl groups.^7^ However, the color and odor of technical lignin hinder the application of lignin for sunscreen preparation.^8^ Currently, solvent fractionation, enzymatic treatment, and chemical modification techniques are used to produce light-colored and odorless lignin. For instance, Lee Sc *et al.* (2020)^9^ studied the potential of cellulolytic enzyme treatment and solvent extraction of rice husk under mild conditions to produce light-colored lignin and nano-lignin, respectively, for sunscreen application. The reported result showed that the obtained lignin and nano-lignin were whiter-colored than conventional lignin. In the study, the sun protection factor (SPF) of lignin-blended sunscreen was also investigated, and it was reported that light-colored nano-lignin showed a higher sun protection factor than light-colored micro lignin. Zhang H *et al.* (2019)^10^ used the solvent fractionation (methanol/water) technique, followed by acetylation (acetic anhydride), to obtain white-colored lignin from black kraft lignin and to investigate its UV absorbability for sunscreen application. The reported result revealed that the approach not only reduced the color of lignin but also enhanced the UV absorbability of lignin, allowing it to synergize with commercial sunscreen (CSS) actives. Xu Y *et al.* (2023)^11^ isolated light-colored lignin from bamboo shoot shells using a hydrated deep eutectic solvent prepared from formic acid, benzyl triethylammonium chloride, and water for sunscreen application. The hydrated deep eutectic solvent (DES) containing 30% water (H_2_O) demonstrated efficient delignification (82.9%), with protected β-O-4 linkage from excessive cleavage. Besides, the isolated lignin exhibited good antioxidant performance compared to the control, and incorporating 5% lignin into a CSS showed a notable improvement in the SPF value. However, despite the potential of isolating light-colored lignin, those methodologies are energy-intensive, and the process takes a long time. In addition, the structure of lignin is highly affected by the isolation method, which leads to the degradation of the aromatic functional groups. Furthermore, particle size has a significant impact on the application of lignin for sunscreen.^3,12^ Ultrasound-assisted deep eutectic solvent (DES)-mediated lignin isolation has the potential to isolate nano-lignin effectively, with a better odor, color, and structure than those from conventional methods in a short time.^13,14^ For instance, Srinivasan S *et al.* (2024)^15^ studied the preparation of lignin, cellulose, and hemicellulose nanoparticles *via* ultrasonication at a frequency of 20 kHz for 15 min using the fractionated ethanol suspensions of the lignocellulosic components. The fractionation of the lignocellulosic components, lignin, cellulose, and hemicellulose, was conducted *via* ultrasound-assisted DES treatment at a sonication frequency of 20 kHz. The reported result showed that ultrasound-assisted DES-mediated treatment was an effective and green technique for preparing nanoparticles with high quality and thermal stability. However, in the study, ultrasonication was conducted twice, first for the fractionation of the lignocellulosic components and second for the nanoparticle preparation. In addition, the color of the obtained nanoparticles was not investigated. Recently, Xie J *et al.* (2026)^16^ investigated the direct isolation and characterization of light-colored lignin nanoparticles from *Pinus radiata* using a ternary deep eutectic solvent (TDES) prepared from benzyltrimethyl ammonium chloride (BTMAC), formic acid (FA), and maleic acid (MA). The study determined that DES-mediated lignin nanoparticle isolation was effective regarding the purity, yield, and color of the lignin nanoparticles, with particle sizes between 127 nm and 171 nm. However, integrating DES with ultrasonication has the potential to reduce the particle size further, with high purity, yield and color. To the best of our knowledge, there is no research studies on the direct isolation of nano-lignin from lignocellulosic biomass using ultrasound-assisted DES-mediated techniques.

Thus, this study aimed to isolate nano-lignin *via* a one-step ultrasound-assisted DES-mediated technique from *Prunus persica* endocarp and characterize its properties such as color, particle size, stability, and structure. Furthermore, this study is intended to investigate the potential of nano-lignin as a sunscreen active to prepare a broad-spectrum sunscreen for protecting skin from a wide range of ultraviolet rays (UVA_1_ from 340 to 400 nm and UVA_2_ from 315 to 340 nm). Thus, following nano-lignin isolation and characterization, a broad-spectrum lignin-blended sunscreen (BSLBS) is prepared by blending nano-lignin (UDES-NL) and a commercial moisturizing sunscreen (SPF 27). The properties of the prepared sunscreen, such as UV absorbance, transmittance, sun protection factor (SPF), rheology, functional groups, color, and anti-microbial activities, are studied to determine the quality of the prepared sunscreen.

## 2 Materials and methods

### 2.1 Materials

The raw material (*Prunus persica* endocarp) was collected from the Holeta Agricultural and Research Center (HARC), Ethiopia. The chemicals, choline chloride (ChCl) and lactic acid (LA), were purchased from Research Product Center (RPI), USA, and HiMedia, India, respectively, for DES preparation. In addition, benzene and ethanol (96%) obtained from HiMedia, India, were used for dewaxing and as a solvent to reduce the viscosity of DES-lignin solution, respectively. The equipment, a jaw crusher (RETSCH, BB 300), sieve shaker (ENDECOTTS, EFL 2000), with an ISO 3310-1 sieve screen, ultrasound homogenizer (BANDLEN UW 3100), and freeze dryer (Lablyo Plus), was used for raw material preparation, nano-lignin isolation, and nano-lignin drying. Zetasizer Ver. 8.02 (ZEN3600, Malvern PANalytical, Malvern, UK) was used for characterizing the particle size and zeta potential of nano-lignin. However, a UV spectrophotometer (UV-vis NI CLB, V-770), Rheometer (Anton Paar), FTIR spectrometer (Nicolet IS50, Thermo Fisher, USA), and spectrophotometer CM-600d (KONICA MINOLTA, INC, JAPAN) were used to characterize the absorption/transmittance, flow property, functional group, and color of sunscreen, respectively.

### 2.2 Methods

#### 2.2.1 Ultrasound-assisted DES-mediated nano-lignin isolation

**Fig. 1:**
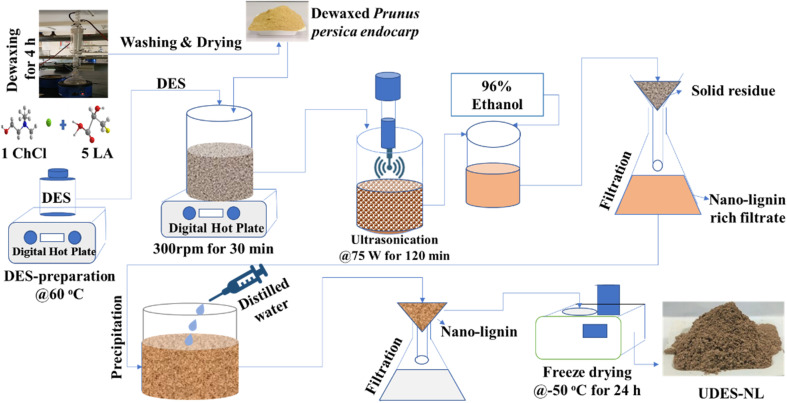
Process flow diagram of ultrasound-assisted DES-mediated nano-lignin isolation.

The raw material, *Prunus persica* endocarp powder, with a 150 µm particle size, was dewaxed using an ethanol and benzene solution (1 : 2 v/v) in a solid to liquid ratio of 1 : 35 (w/v) of solid loading for 4 h.^17^ Then, the dewaxed powder was washed and dried for the nano-lignin isolation. The nano-lignin isolation was carried out *via* ultrasound-assisted DES (ChCl : LA, 1 : 5 molar ratio) treatment. The ultrasound-assisted DES-mediated nano-lignin isolation was conducted using dewaxed *Prunus persica* powder at 7% solid loading, 120 °C, 120 min, and 75 W sonication power. Following sonication, 96% ethanol was added to the sonicated mixture in 1 : 2 v/v as a cosolvent to reduce the viscosity of the sonicated solution and enhance filtration. In general, adding aqueous ethanol and acetone is helpful to solvate the hydrophobic and hydrophilic motifs of lignin. The solid residue in the sonicated solution was first separated by filtration. During filtration, ethanol was added to enhance the filtration, *i.e.*, to increase the amount of the filtrate. In the filtrate, a two-fold amount of distilled water was added until precipitating particles were seen, and it was left for some time. Subsequently, the precipitated solution was separated by filtration, and the solid particle (named nano-lignin (UDES-NL)) was trapped on a filter paper, and it was washed several times by adding distilled water until a neutral pH was achieved. Finally, the solid particle was freeze-dried at −55 °C for 24 h.^18^ The overall process flow diagram of the nano-lignin isolation is described in Fig. 1.

#### 2.2.2 Preparation of the broad-spectrum lignin-blended sunscreen (BSLBS)

**Fig. 2:**
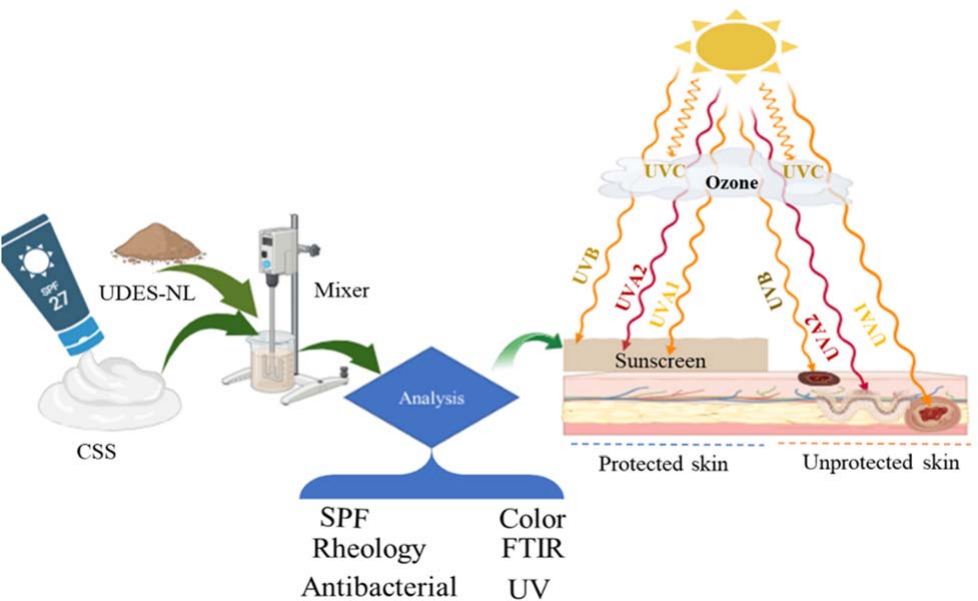
Flow diagram of the BSLBS preparation and schematic of its impact on the skin.

The preparation of the broad-spectrum lignin-blended sunscreen (BSLBS) was conducted in the dark as follows: the nano-lignin obtained from *Prunus persica* endocarp *via* ultrasound-assisted DES was blended with an SPF-27 moisturizing commercial sunscreen (CSS) under magnetic stirring (600 rpm, 3 h) at different weight percentages (1 wt%, 5 wt%, and 10 wt%) of the total weight of 3 g.^7,11,12^ The process flow diagram and schematic of the sunscreen preparation and application are shown in Fig. 2.

#### 2.2.3 Characterization of nano-lignin obtained *via* ultrasound-assisted DES (UDES-NL)

##### 2.2.3.1 Determination of the yield and purity of UDES-NL

The yield of UDES-NL was determined using eqn (1): the amount of UDES-NL divided by the initial amount of lignin in the biomass sample (53.98%). The purity of UDES-NL was determined by adding acid-soluble nano-lignin (ASNL) and acid-insoluble nano-lignin (AINL) *via*eqn (5).^19,20^ A UV-vis spectrophotometer (UV-vis NIR CLB, V-770) was used to measure the absorbance of UDES-NL at 205 nm.^20^ The amounts of ASNL and AINL were quantified using eqn (3) and (4), respectively.1
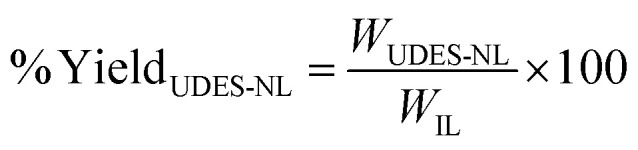
2
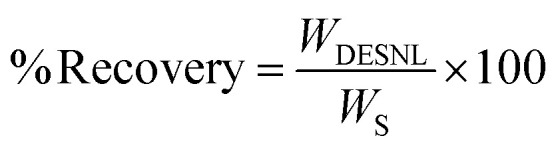
3
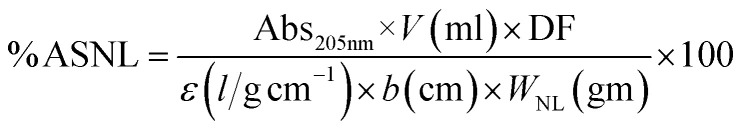
4
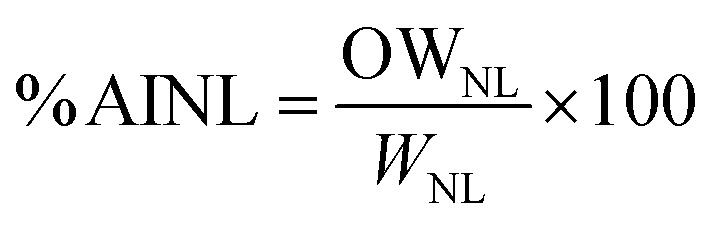

5 Purity = ASNL + AINL

where UDES-NL is the nano-lignin obtained *via* ultrasound-assisted DES, *W*_UDES-NL_ is the weight of UDES-NL, *W*_IL_ is the initial weight of lignin in the biomass, *W*_S_ is the weight of the biomass sample, and ASNL is the acid-soluble nano-lignin. Abs_205 nm_ is the absorbance of nano-lignin at 205 nm, *V* is the volume of filtrate, DF is the dilution factor, *ε* is the absorptivity at a specific wavelength (110 L g^−1^ cm^−1^), *b* is the optical path length and *W*_NL_ is the dry weight of nano-lignin. AINL is the acid-insoluble nano-lignin, OW_NL_ is the oven-dry weight of hydrolyzed nano-lignin, and *W*_NL_ is the dry weight of nano-lignin.

##### 2.2.3.2 Particle size and stability analysis of UDES-NL

The particle size and zeta potential of UDES-NL were determined using Zetasizer.Ver.8.02 (ZEN3600, Malvern Panalytical, Malvern). The sample holder's disposable micro cuvette and disposable clear zeta cell were used for particle size and zeta potential analyses, respectively. The analysis was conducted at a scattering angle of 173°, dispersant refractive index (RI) of 1.333, viscosity of 0.8872 cp and dispersant dielectric constant of 78.5. The analysis was carried out at a concentration of 0.1 mg mL^−1^ by mixing nano-lignin with 10 mL of deionized water and homogenizing using an ultrasound for 10 min.^12^

##### 2.2.3.3 Structural analysis of UDES-NL

The structural analysis of UDES-NL was performed using an X-ray diffractometer (XRD, Rigaku Ultima IV, Rigaku), working at 40 kV and 44 mA. XRD analysis was conducted to assess whether the nanoparticle synthesis process induced any modifications to the intrinsic amorphous structural nature of nano-lignin or not over a 2*θ* range of 10°–80°, using CuKα radiation (*λ* = 1.54060 Å).^12^

#### 2.2.4 Characterization of the broad-spectrum lignin-blended sunscreen (BSLBS)

##### 2.2.4.1 Functional group analysis

The functional groups of UDES-NL and BSLBS were determined by FTIR, scanning the samples from 4000 to 400 cm^−1^.^9^

##### 2.2.4.2 Optical properties

The optical property analyses of DES-NL and BSLBS were conducted using spectrophotometry (CM-600d, KONICA MINOLTA, INC., Japan). Color was measured using the L*, a*, b* color space, where L* represents brightness, L* = 100 indicates white, and L* = 0 indicates black. +a* is the red direction, and −a* is the green direction; +b* is the yellow direction, and −b* is the blue direction. The total color difference (Δ*E*) was obtained directly from the instrument, which could also be calculated using eqn (6).^21^6
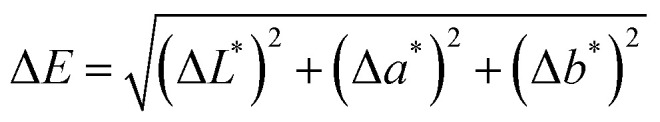


##### 2.2.4.3 UV-absorbance and UV-transmittance analyses

The UV-absorbance and transmittance properties of UDES-NL and BSLBS were determined using a UV spectrophotometer (UV-vis NIR CLB, V-770) equipped with a solid sample holder. The scanning was performed from 200 nm to 800 nm for absorbance and 290 nm to 400 nm for transmittance.^9^

##### 2.2.4.4 Sun protection factor (SPF) determination

*In vitro* sun protection factor determination was conducted using UV transmission and eqn (7).^9^ The sample preparation for the determination of the sun protection factors for each type of prepared sunscreen (1% UDES-NL, 5% UDES-NL and 10% UDES-NL) and the control CSS was conducted separately as follows: 3M Transpore tape was plastered on quartz slides, 2 mm wide. A tape of 12.5 cm^2^ was used to ensure that measurements were carried out on at least five non-overlapping spots. For a 12.5 cm^2^ sample size, the slide was placed on an analytical balance, and 2 mg cm^−2^ of BSLBS was distributed on the tape by dotting sunscreen on the slide. The sunscreen was then distributed over the entire surface by slowly rubbing the slide surface with a cot-coated finger. The sample was then placed in a dark room and dried for 20 min prior to measurement. The measurements were conducted for a 1-nm wavelength step.^12^7
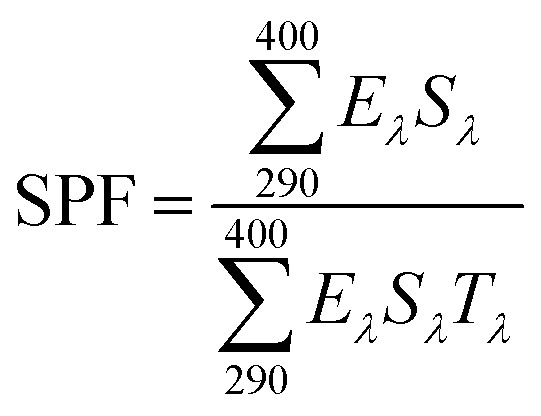
where *E*_*λ*_ is the erythemal spectral effectiveness (CIE-1987),^22^*S*_*λ*_ is the solar spectral effectiveness,^22^ and *T*_*λ*_ is the spectral transmittance of the sample.

##### 2.2.4.5 Rheological property analysis

The rheological properties, such as viscosity, shear stress, and shear rate, of the prepared sunscreen (BSLBS) were determined *via* a rheometer (ANTON PAAR RHEO COMPASS) at a temperature of 25 °C.^23^

##### 2.2.4.6 Antimicrobial activity analysis

The antimicrobial activity analysis of UDES-NL, sunscreen, and UDES-NL-blended sunscreen was conducted. For the analysis, strains of *Escherichia coli* (*E. coli*) and *Staphylococcus aureus* (*S*. *aureus*) were freshly grown at 37 °C for 24 h in a nutrient broth. To evaluate the sensitivity of UDES-NL, sunscreen, and UDES-NL-blended sunscreen against bacterial strains, a well-diffusion assay was conducted by carefully spreading 0.1 mL of bacterial culture on the Mueller–Hinton agar (MHA) surface. Eight wells were made on each MHA plate, and the wells contained 30 µL of CSS blended with different amounts of UDES-NL (1%, 5%, and 10%). CSS and ciprofloxacin were used as negative (−) and positive (+) controls, respectively. The plates were incubated at 37 °C for 24 h, and the inhibition zones were then measured. The antibacterial effectiveness of UDES-NL containing CSS was assayed using 1 cm^2^ of UDES-NL containing sunscreen. The sunscreen was applied to the MHA surface containing bacteria and incubated at 37 °C for 24 h. After incubation, the zone of inhibition was measured.^24^

## 3 Results and discussion

### 3.1 Characterization of the nano-lignin isolated *via* ultrasound-assisted DES (UDES-NL)

#### 3.1.1 Analysis of the yield, purity, particle size, stability, and morphology of UDES-NL

##### 3.1.1.1 Analysis of the yields and purity of UDES-NL

**Fig. 3:**
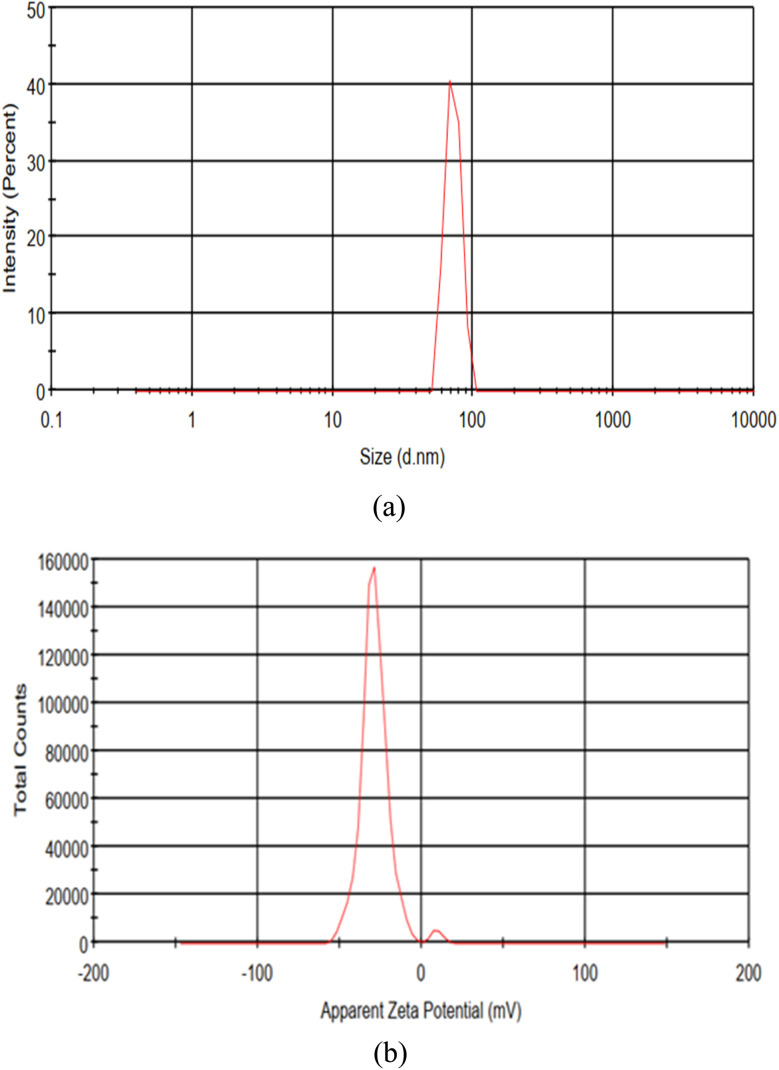
Particle size (a) and zeta potential (b) analyses of UDES-NL.

The yield of the nano-lignin obtained *via* ultrasound-assisted DES-mediated isolation (UDES-NL) was determined to be 75.58% using eqn (1). However, the purity of UDES-NL was quantified after obtaining ASNL and AINL using eqn (4). ASNL and AINL were obtained at 4.61% ± 0.093% and 92.67% ± 0.471%, respectively. Thus, the purity of UDES-NL was obtained at 97.27%, which was the sum of ASNL and AINL. Previously,^20^ lignin nanoparticles were isolated directly from *Pinus radiata* using a ternary deep eutectic solvent (TDES) prepared from formic acid (FA), maleic acid (MA), and benzyl-trimethyl-ammonium chloride (BTMAC). The reported result showed that the yields of the lignin nanoparticle was 84.7%, with a purity of 94.1%. Comparing the yields of UDES-NL of this study with the reported literature values, the yields of UDES-NL of this study were lower, while the purity was higher. The decrease in the UDES-NL yield could be due to filtration during the separation of UDES-NL from solid residue and the filtration of precipitated UDES-NL to separate it from DES and excess water. In these stages, UDES-NL could be trapped on a filter paper and remain in the solid residue. Thus, to increase the yields, carefully separating UDES-NL from the solid residue by repeatedly adding ethanol would be important. In addition, using centrifugation rather than the filtration of precipitated UDES-NL would also increase the yield of UDES-NL. However, ultrasound-assisted DES-mediated nano-lignin isolation afforded the purest and light-colored nanoparticles, with a small particle size, 75.38 nm, as shown in Fig. 3(a). Thus, this technique could be considered more effective than conventional nanoparticle preparation methods, as shown in Table 1. However, a recovery rate of 40.8% was obtained, indicating that further research work is required to investigate the most effective nano-lignin isolation technique and implement the biorefinery concept for the valorization of lignocellulosic components.

**Table 1:** Summary of comparison of UDES-NL yield and purity with literature values

Material	Technique	Parameters	Yield (%)	Purity (%)	Particle size (nm)	Color	Ref.
Rice husk	Solvent shifting and sonication of lignin	THF-water mixture, 7 : 3(v/v), sonication power of 150 W, 20 min	—	90	225	Lightness (L*) of 66.8	9
*Sorghum*	Two steps: isolation of lignin *via* DES + ultrasonication and ultrasonication lignin to nano-lignin	20 kHz, 15 min	—	—	57.2	—	15
*Pinus radiata*	One step using ternary DES	130 °C, 1.5 h	84.7	94.1	127–171	Light colored (ISO 24.5%)	16
*Prunus persica*	One-step ultrasound + DES	120 min, 120 °C, and 75 W of sonication	75.8	97.27	75.38	Lightness (L*) of 57.65	This study

##### 3.1.1.2 Particle size and stability analysis of UDES-NL

The particle size and stability of UDES-NL were determined using a zeta sizer; the numerical results obtained are shown in Tables 2 and 3, respectively. The plots of the particle size are shown in Fig. 3(a) and (b) presents the stability or zeta potential. In Fig. 3, the attained particle size and zeta potentials of UDES-NL are presented. As shown in Fig. 3(a), the average particle size of 75.38 nm, with a polydispersity index (PDI) value of 0.663, of UDES-NL was obtained, which indicated a uniform size distribution. Correspondingly, in Fig. 3(b), the zeta potential results of UDES-NL, with a value of −28.2 mV, are presented. Zhang *et al.*^25^ studied the properties of lignin nanoparticles, including stability and particle distribution. Nanoparticles were prepared from alkali lignin using ethyl glycol with subsequent ultrasonication and dialysis. The reported result showed that the zeta potentials of the nanoparticle were between −20 mV and −40 mV and stable over a pH range of 3–12 in aqueous solution. The pH of the human skin is generally between 4.5 and 6.0.^26^ Correspondingly, most sunscreens are formulated to match the pH of the human skin. Therefore, the nano-lignin obtained in this study, *i.e.*, UDES-NL, having a zeta potential of −28.2 mV, can be utilized as a sunscreen active.

**Table 2:** Particle size analysis results derived from the zeta sizer

Analysis	Peak	Size (*d* nm)	Intensity (%)	St. Dev. (*d* nm)	Average particle size (*d* nm)	PDI	Intercept
Particle size	1	72.26	100.0	1.334	75.38	0.66	1.03
	2	0.00	0.00	0.00			

**Table 3:** Stability analysis results derived from the zeta sizer

Analysis	Peak	Mean (mV)	Area (%)	St. Dev. (mV)	Zeta potential (mV)	Zeta deviation (mV)	Conductivity (mS cm^−1^)
Stability	1	−28.9	98.2	8.07	−28.2	9.49	0.204
	2	9.13	1.8	3.51			

The negative zeta potential values indicate electrostatic repulsion between the particles, which enhances dispersion stability *via* the electric double layer (EDL), though stability also depends on ionic strength and other interactions. Nano-lignin contains acidic groups, such as phenolic and carboxylate hydroxyl functional groups, on its surface, which give nano-lignin a negative surface charge. Under low acidic conditions, carboxyl and phenol groups remain protonated, affording a low negative surface charge and weak EDL. Thus, as seen in Fig. 5, the FTIR analysis revealed that UDES-NL contained phenolic hydroxyl and carboxyl functional groups, which resulted in a negative surface charge on UDES-NL. However, in terms of particle size, a large surface area enhanced the UV-absorbing activity of nano-lignin.^27^

Where *d* nm is the diameter in nanometers, and St. Dev. is the standard deviation as obtained using the instrument.

Where mV is millivolt, St. Dev. is the standard deviation as obtained from the instrument, and mS cm^−1^ is millisiemens per centimeter.

##### 3.1.1.3 X-ray diffraction (XRD) analysis of UDES-NL

**Fig. 4:**
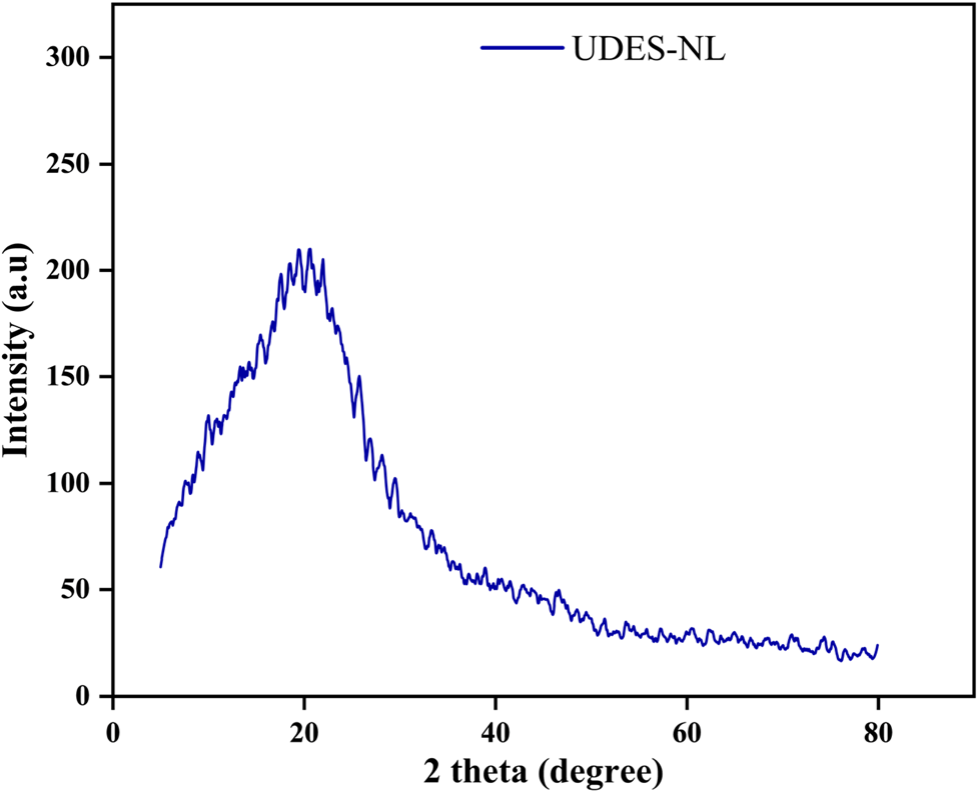
X-ray diffraction pattern of UDES-NL.

The analysis of UDES-NL *via* XRD was conducted to determine if the amorphous nature of nano-lignin was preserved. In Fig. 4, the XRD plot of UDES-NL is shown. As seen in the plot, a broad, intense peak was found at 19.41°. This broad peak was characteristic of amorphous materials, confirming the amorphous nature of the nano-lignin obtained *via* the ultrasound-assisted DES-mediated (UDES-NL) technique. The nonappearance of further sharp diffraction peaks indicated that the preparation process did not incorporate any crystalline phase into the nano-lignin structure.^12^ Thus, the XRD analysis revealed that the isolation of nano-lignin *via* the ultrasound-assisted DES-mediated technique preserved the intrinsic amorphous nature of nano-lignin.

### 3.2 Analysis of the properties of the broad-spectrum lignin-blended sunscreen (BSLBS)

#### 3.2.1 Fourier transform infrared (FTIR) spectroscopy analysis

**Fig. 5:**
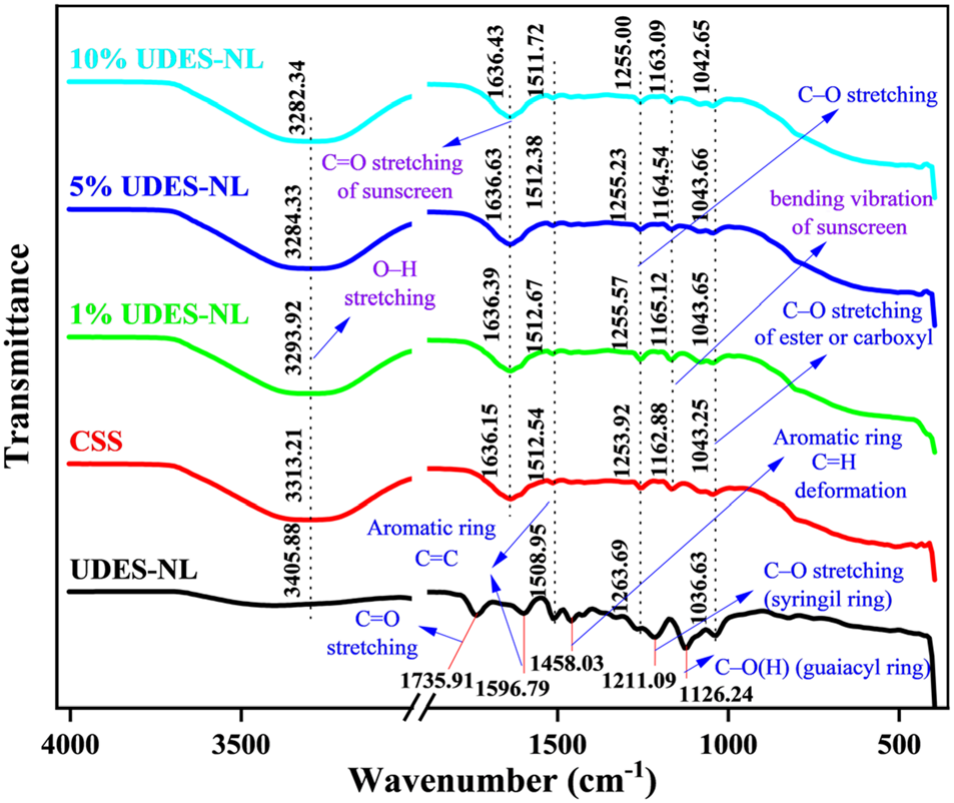
FTIR analysis of UDES-NL, CSS, and their blends: 1%, 5%, and 10% of UDES-NL.

FTIR analysis was conducted to determine the functional groups, intermolecular interaction of lignin and sunscreen actives, stability of the prepared sunscreen, and presence of UV-absorbing functional groups in the formulated sunscreen. Thus, as shown in Fig. 5, the FTIR analysis was performed for the nano-lignin isolated *via* ultrasound-assisted deep eutectic solvent (UDES-NL), CSS, and their blends at 1% UDES-NL, 5% UDES-NL, and 10% UDES-NL to form a broad-absorption-spectrum sunscreen. The FTIR spectrum of the sunscreen showed the presence of organic UV filters and additives. The broad peak of the sunscreen observed at 3313.21 cm^−1^ (O–H stretching) indicated the presence of glycerol- or alcohol-based moisturizing ingredients. Peaks observed at 1636.15 cm^−1^ due to C=O stretching and 1162.88 cm^−1^ due to bending vibration indicated the presence of benzophenone- and cinnamate-based organic UV filters in the sunscreen.^28^ In addition, very short intensity peaks observed at 1512.54 cm^−1^ indicated the C=C stretching of an aromatic ring, whereas 1253.92 and 1043.25 cm^−1^ corresponded to C–O stretching.

The FTIR spectrum of UDES-NL showed the presence of phenolic, aromatic, syringyl, and guaiacyl functional units. The peak formed at 3405.88 cm^−1^ indicated the O–H stretching of a phenolic hydroxyl group. The peak at 1732.04 cm^−1^ corresponded to the C=O stretching bond of a carboxyl group. The main characteristic peaks, 1596.79, 1508.95, and 1427.08 cm^−1^, represented aromatic ring vibration.^29,30^ The absorption peak at 1458.03 cm^−1^ assigned to C–H deformation demonstrated the occurrence of an aromatic hydroxyl group. The peaks at 1263.69, 1211.06, and 1126.24 cm^−1^ were the C–O stretching of the ester bond in syringyl and guaiacyl units. However, the ester bond at 1033.49 cm^−1^ was assigned to the C–H deformation of guaiacyl unit.^29^ The presence of syringyl and guaiacyl promoted the UV shielding performance of UDES-NL.^27^ In the FTIR spectrum of the CSS and UDES-NL blend (1% UNDES-NL, 5% UNDES-NL, and 10% UNDES-NL), the peaks were formed between the peaks of sunscreen (CSS) and UDES-NL. The peak shifts and intensity variations shown in the spectrum of CSS and UDES-NL blend could be due to sunscreen or UDES-NL. For instance, the O–H stretching bands of 1% UDES-NL, 5% UDES-NL, and 10% UDES-NL were formed between 3405.88 cm^−1^ of UDES-NL and 3313.21 cm^−1^ of sunscreen. As a result, unexpected new peaks were not formed, indicating the blending process did not cause chemical degradation or undesirable side reactions. The observed peaks indicated promising physical interactions and compatibility between CSS and UDES-NL. However, the retained aromatic and phenolic functionalities of UDES-NL revealed that its UV-absorbing potential was preserved, confirming that ultrasound-assisted DES-mediated nano-lignin isolation was a promising approach.

#### 3.2.2 Optical property analysis

The optical properties of the nano-lignin isolated *via* ultrasound-assisted deep eutectic solvent (UDES-NL) were determined using spectrophotometry (CM-600d, KONICA MINOLTA, INC., Japan), as indicated in Table 4. Before analysis, the optical property of the sample holder (white reference) was determined, and 99.0 lightness (L*), with a color change (Δ*E*) value of 50.54, was obtained. The obtained result showed that UDES-NL had a lightness (L*) value of 57.65 with a 25.05 color-change value. The obtained lightness index value of UDES-NL indicated that its color was mild white. Thus, this helped to prepare sunscreens of different colors by varying the amount of UDES-NL for different skin tones. The lightness values revealed that the ultrasound-assisted deep eutectic solvent-mediated nano-lignin isolation was a promising technique to obtain nano-lignin suitable for sunscreen application.

**Table 4:** Color change and color scheme of nano-lignin (UDES-NL), CSS and mixture of UDES-NL and sunscreen

Sample	L*	a*	b*	ΔL*	Δa*	Δb*	Δ*E*
White reference	99.40 ± 0.045	−0.12 ± 0.006	−0.04 ± 0.002	63.49 ± 3.174	0.69 ± 0.0345	0.23 ± 0.0115	50.54 ± 1.243
UDES-NL	57.65 ± 2.882	6.76 ± 0.338	13.68 ± 0.684	21.75 ± 1.0875	7.57 ± 0.3785	13.95 ± 0.697	25.05 ± 2.505
CSS	80.81 ± 4.040	−1.72 ± 0.086	6.65 ± 0.332	44.90 ± 2.245	−0.91 ± 0.045	6.92 ± 0.346	40.89 ± 4.089
1% UDES-NL	63.84 ± 3.192	4.42 ± 0.221	10.53 ± 0.526	27.94 ± 1.397	5.24 ± 0.262	10.80 ± 0.54	29.93 ± 2.993
5% UDES-NL	46.19 ± 2.309	5.74 ± 0.287	9.10 ± 0.455	10.29 ± 0.514	6.55 ± 0.3275	9.37 ± 0.4685	14.30 ± 1.43
10% UDES-NL	39.13 ± 1.956	5.51 ± 0.275	7.67 ± 0.383	3.22 ± 0.161	6.32 ± 0.316	7.94 ± 0.397	10.59 ± 1.059

**Fig. 6:**
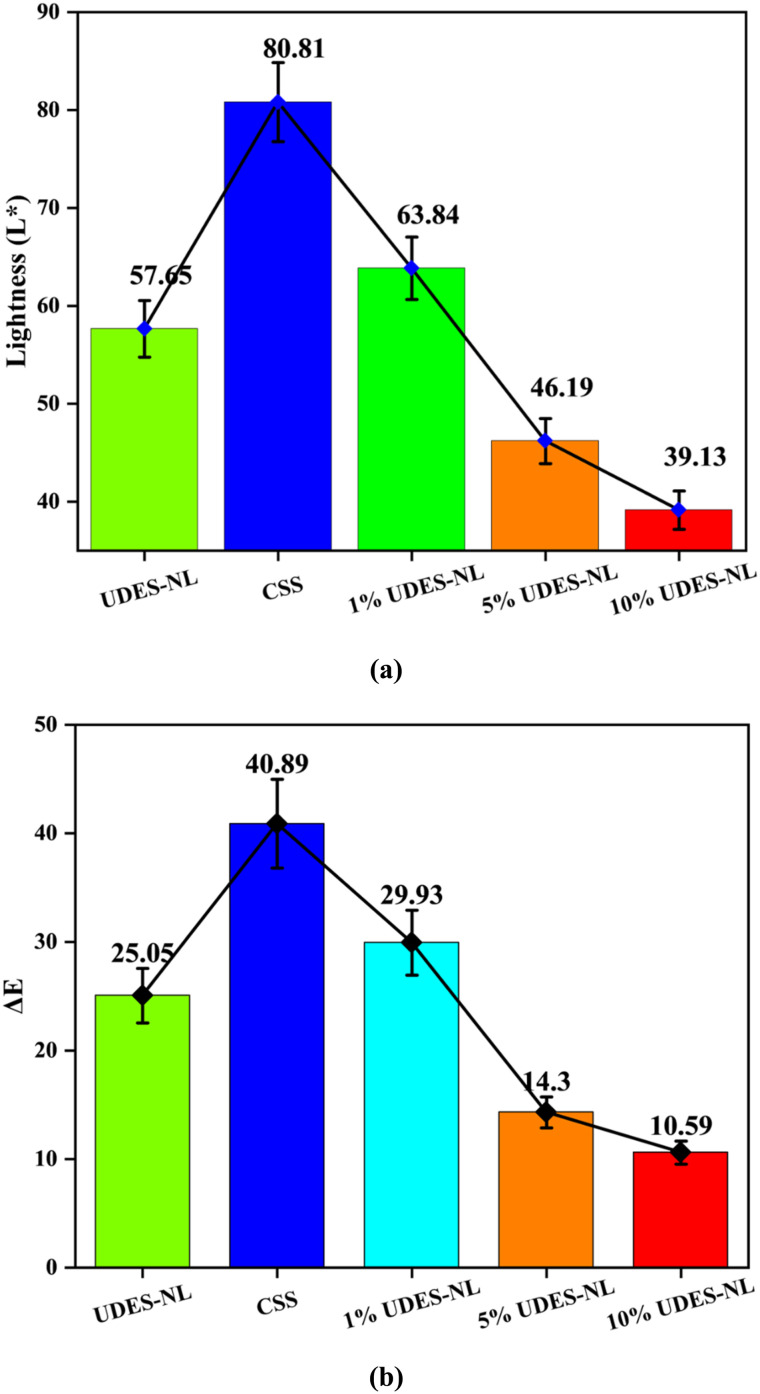
Lightness (L*); (a) and color change (Δ*E*) analyses; (b) of the nano-lignin obtained *via* ultrasound-assisted deep eutectic solvent (UDES-NL), CSS, and blends of sunscreen and UDES-NL.

Thus, the optical property of the broad-spectrum nano-lignin-blended sunscreen (BSLBS) prepared from nano-lignin and CSS was determined, as shown in Table 4. The analysis showed that the sunscreen had the lightness index values of 80.81, with a 40.89 color change (Δ*E*) value. However, the lightness index values of BSLBS with 1% UDES-NL, 5% UDES-NL, and 10% UDES-NL were 63.84, 46.19, and 39.13, respectively, while the corresponding color change values were 29.93, 14.30, and 10.59, respectively. Comparing the optical properties, BSBLS prepared from 1% UDES-NL had the highest lightness index value and the highest color change value. However, BSLBS with 10% UDES-NL had the lowest change in color value and relatively low lightness index value, as depicted in Fig. 6(a) and (b), respectively. Thus, based on the lightness index value and visual inspection, the prepared BSLBS showed different colors: white, light brown, and brown, which could be harmonized with different skin tones.

#### 3.2.3 Analyses of UV-absorbance and UV-transmittance

**Fig. 7:**
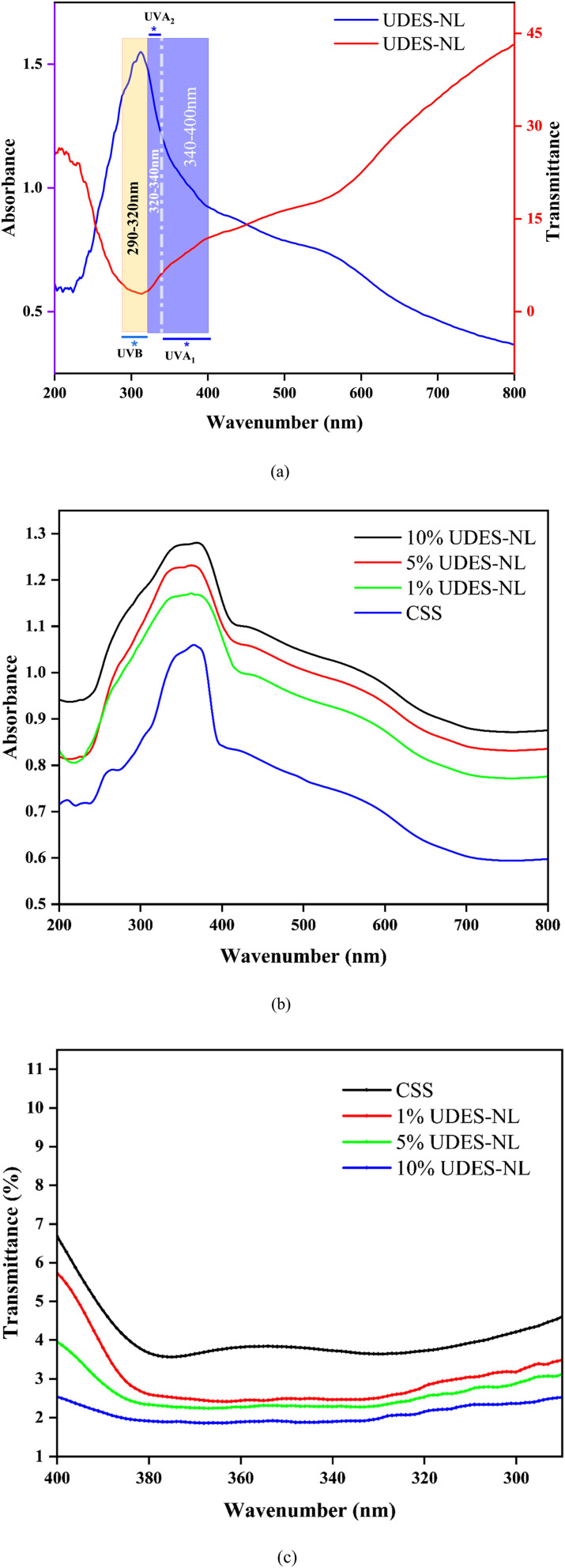
UV absorbance and transmittance spectra of (a) UDES-NL and (b) CSS and BSLBS at different amounts of UDES-NL. (c) Transmittance of CSS and BSLBS at different amounts of UDES-NL.

The absorbance and transmittance of the prepared UDES-NL analysis were conducted from 200 to 800 nm to investigate the potential of the isolated nano-lignin for sunscreen application. In Fig. 7(a), the UV absorbance and transmittance analysis results of nano-lignin isolated *via* ultrasound-assisted deep eutectic solvent (UDES-NL) are described. As shown in Fig. 7(a), UDES-NL formed a sharp peak with a wide-range absorption spectrum, covering UVB, UVA_1_, and UVA_2_, which indicated the existence of chromophores and auxochromic groups, which allowed UDES-NL to be used for UV absorption.^31^ As a result, UDES-NL could be utilized for broad-spectrum sunscreen preparation. Thus, in this study, UDES-NL was blended at different concentrations (1% UDES-NL, 5% UDES-NL, and 10% UDES-NL) with SPF 27 CSS, and the possibilities of UDES-NL for broad-spectrum sunscreen application were investigated. In Fig. 7(b), the absorbance results of CSS (SPF27) and formulated sunscreen are shown. As seen in Fig. 7(b), the absorption capacity of the UDES-NL-blended sunscreen was higher than that of CSS. In addition, it was also shown that when the amount of UDES-NL increased, the absorption efficiency of the sunscreen also increased, which indicated the significance of UDES-NL absorption effectiveness. Fig. 7(c) shows the transmittance of the pure sunscreen and UDES-NL-blended sunscreen from 290 to 400 nm. Ultraviolet radiation in the range between UVB (280–320 nm), UVA_2_ (320–340 nm), and UVA_1_ (340–400 nm) can cause photoaging and skin cancer.^32^ However, incorporating broad UV-absorbing organic materials, such as lignin, has the potential to mitigate the impacts. As seen in Fig. 7(c), the UV transmittance of the UDES-NL-blended sunscreen has reduced slowly between 290 and 400 nm, with a pronounced effect at 10% UDES-NL. As a result, this study confirmed the possibility of preparing a broad-spectrum sunscreen by incorporating nano-lignin into an SPF27 commercial sunscreen.

#### 3.2.4 Sun protection factor (SPF) analysis

**Fig. 8:**
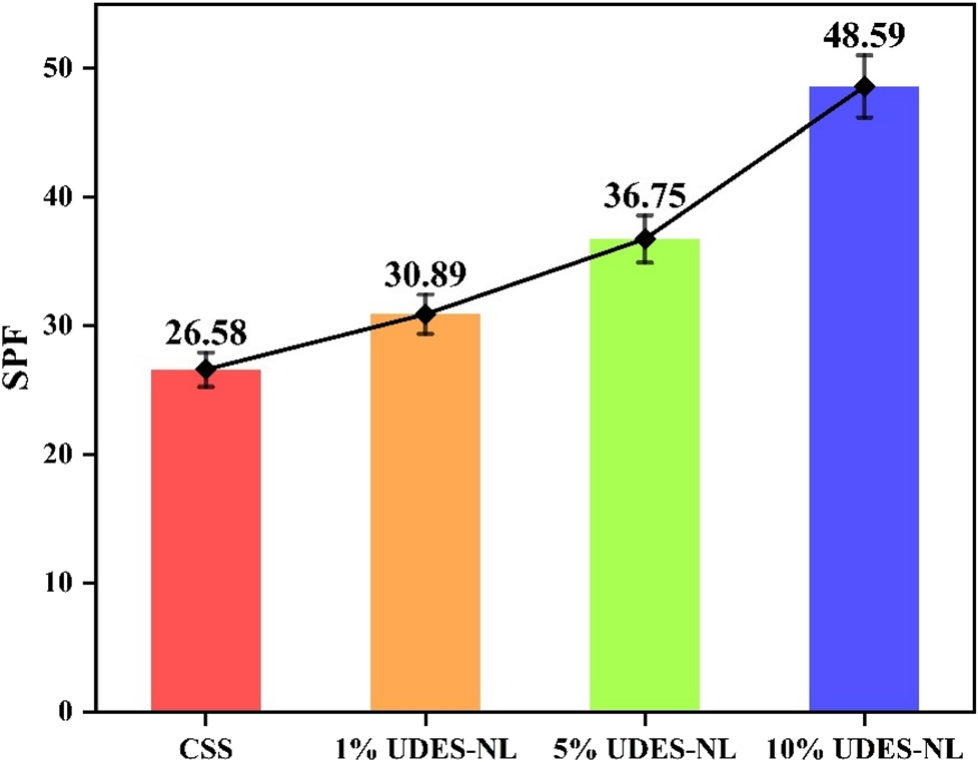
Sun protection factor analyses of CSS and its blends such as 1% UDES-NL, 5% UDES-NL and 10% UDES-NL.

The sun protection factor analysis was conducted to determine the UV protection efficiency of the prepared sunscreen. Thus, the SPF values of CSS and the prepared UDES-NL blended sunscreen were determined from the UV transmittance data, as described in Fig. 7(c). As shown in Fig. 8, the SPF value of CSS alone was increased from 26.58 to 30.89, 36.75, and 48.59 upon the addition of 1% UDES-NL, 5% UDES-NL, and 10% UDES-NL, respectively. This increment in the SPF value with nano-lignin concentration indicated the potential of nano-lignin to enhance UV absorption, leading to reduced UV transmittance across the tested spectrum. This result was in line with the literature value of Yan Z *et al.* (2023),^33^ who studied the effects of nano-lignin on the SPF value of sunscreen obtained from kraft lignin *via* ultrafiltration membrane fractionation. The researchers investigated the increment in SPF value when nano-lignin was added to the sunscreen, and they observed an increase from SPF 15 to SPF 63.74. The observed phenomenon was due to the nano-scale particle size and the resulting surface area of nano-lignin.^21^ In addition, the functional groups, such as hydroxyl, carboxyl, and methoxy groups, in lignin, as shown in Fig. 5, also enhanced the UV absorption of sunscreen.^11^ Overall, based on the SPF values, this study revealed the possibility of preparing a broad-spectrum sunscreen using nano-lignin, with the potential to absorb UV light in the wavelength range from 290 to 400 nm. Furthermore, conventional sunscreens often require a minimum of 20% chemical actives to reach SPF 15 and about 30% to attain SPF 50, potentially leading to the usage of a substantial amount of sunscreen actives, which raises health and environmental concerns. As a result, considering the availability and promising UV-absorbing properties, nano-lignin-containing sunscreens would have the potential to expand the range of everyday skin-care options and would be considered as a future sunscreen active.

#### 3.2.5 Rheological property analysis

**Fig. 9:**
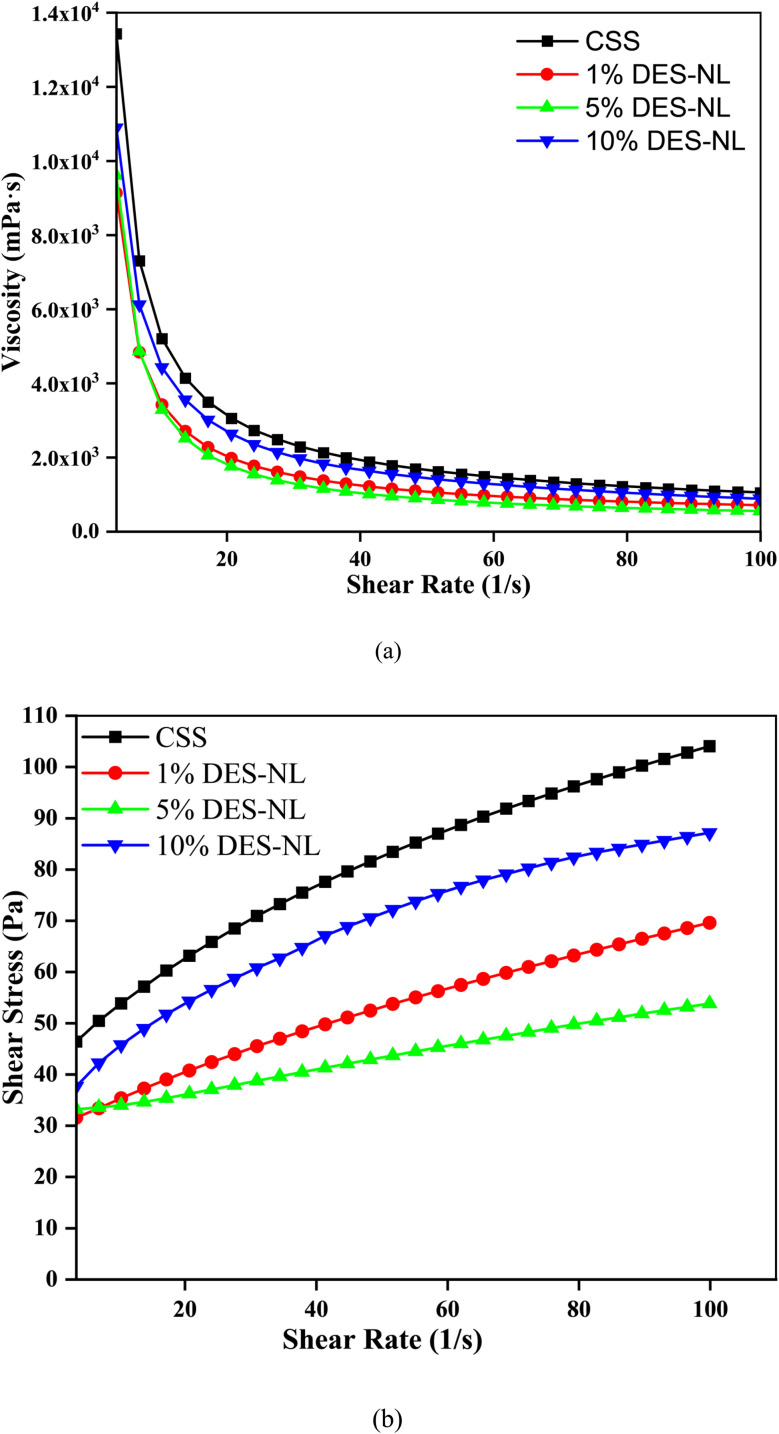
Flow curve of CSS and its blend with UDES-NL at different concentrations for rheological properties analysis (a) viscosity *vs.* shear rate (b) shear stress *vs.* shear rate.

The rheological properties of the pure sunscreen and sunscreen blends containing 1%, 5%, and 10% UDES-NL were characterized by recording viscosity *vs.* shear rate on an Anton Paar rheometer. From the flow curves, shear thinning/thickening properties, flowability, and spreadability were determined using viscosity *vs.* shear rate and shear stress *vs.* shear rate plots, as shown in Fig. 9(a) and (b), respectively. As shown in Fig. 9(a), the viscosities of all samples decreased with increasing shear rate, indicating that the prepared sunscreens were non-Newtonian, shear-thinning fluids. The effect of UDES-NL on the viscosity of the pure sunscreen was investigated at a constant shear rate of 3.45 s^−1^. The addition of 1%, 5%, and 10% UDES-NL reduced the viscosity of CSS from 13427 mPa s to 9127.5 mPa s, 9598.7 mPa s, and 10892 mPa s, respectively, indicating improved flowability. The rheological analysis suggested that the prepared sunscreen exhibited properties favorable for skin application (easily spread when applied) and potential for enhanced transportability during storage due to increased thickening.

The Herschel–Bulkley equation, as defined in eqn (3), was used for determining the values of the flow behavior index, consistency index, and yield stress, which helped to further investigate the rheological properties, shear thinning or shear thickening behavior, flowability, and spread ability.^34^

The yield stress (*τ*_y_) signifies the initial stress to start flow, which determines the internal structural strength of a sunscreen.^34^ Thus, a higher yield stress means better stability and resistance to dripping, which is essential for sunscreen preparation, confirming sunscreen uniformity and keeping runoff after use. The consistency index (*K*) implies the thickness of a sunscreen. A higher consistency value means a thicker or creamier texture, while a lower consistency signifies a lighter, more spreadable sunscreen.^35^ Further, the flow index (*n*) describes the degree of non-Newtonian behavior; values of *n* < 1 denote shear-thinning behavior, and *n* > 1 denotes shear thickening, which enables easy spreading for use and recovery to a stable structure at rest.^35^ As shown in Table 5, the flow index values were between one and zero, *i.e.*, 0.559 ± 0.012, 0.725 ± 0.006, 0.969 ± 0.022, and 0.441 ± 0.022 for sunscreen, 1% UDES-NL, 5% UDES-NL, and 10% UDES-NL, respectively, *i.e.*, their fluid properties were shear thinning (pseudoplastic). However, the yield stress values of 34.612 ± 0.803, 27.309 ± 0.181, 31.577 ± 0.208, and 21.381 ± 1.993 were obtained for sunscreen, 1% UDES-NL, 5% UDES-NL, and 10% UDES-NL, respectively. The yield stress values (*τ*_y_) were greater than zero. Thus, Herschel–Bulkley model equation could describe the flow properties of CSS and blended sunscreen. Lastly, the consistency index (*K*) values of 5.323 ± 0.342, 15.704 ± 1.696, 20.695 ± 2.925, and 22.621 ± 3.040 were obtained for sunscreen and for 1% UDES-NL, 5% UDES-NL, and 10% UDES-NL blended sunscreens, respectively. The result indicated that the sunscreen alone was lighter than the UDES-NL-blended sunscreen. These rheological parameters provided insight into the flowability, spreadability, and stability of sunscreen. In line with the results of this study, the rheological property analysis of sunscreens has also been described in previous studies.^34,35^

9 *τ* = *τ*_y_ + *Kγ*^*n*^

where *τ* is stress (Pa), *γ* is the shear rate (s^−1^), *τ*_y_ is the initial stress (Pa), *K* is the consistency index (Pa s^*n*^), and *n* is the flow index, which are the fitting parameters.

**Table 5:** Herschel–Bulkley parameters for the original sunscreen and the sunscreen blended with UDES-NL obtained after fitting, as presented in Fig. 9(b)^a^

*τ*_o_ (Pa)	*K* (Pa s^*n*^)	*n* (dimensionless)	*R* ^2^	Product
34.612 ± 0.803	5.323 ± 0.342	0.559 ± 0.012	0.999	Sunscreen
27.309 ± 0.181	1.502 ± 0.049	0.725 ± 0.006	0.999	1% UDES-NL
31.577 ± 0.208	0.258 ± 0.027	0.969 ± 0.022	0.998	5% UDES-NL
21.381 ± 1.993	8.771 ± 1.121	0.441 ± 0.022	0.997	10% UDES-NL

a The values after ± are standard errors, not standard deviation, which are directly obtained from regression analysis.

#### 3.2.6 Antimicrobial property analysis

**Fig. 10:**
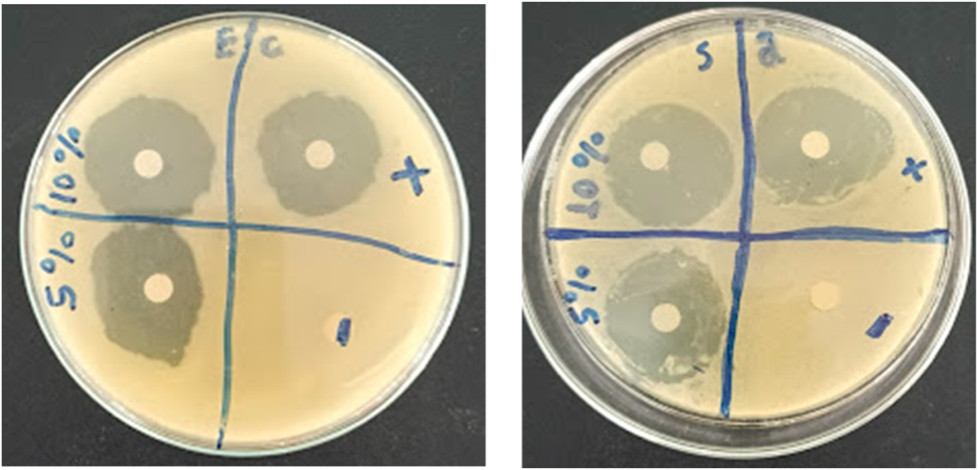
Antimicrobial activity of CSS and sunscreen blended with 1%, 5%, and 10% UDES-NL against *E. coli* (E. c), and *Staphylococcus aureus* (S. a) bacteria; ciprofloxacin is taken as the +ve control and CSS alone is taken as the −ve control. Note: diameters of the shortest radius were reported for oval-shaped zones.

The antimicrobial activities of CSS and sunscreen blended with different amounts of UDES-NL against microbial strains, *E. coli* and *Staphylococcus aureus*, were determined using the well-diffusion technique, as shown in Fig. 10, and the measured values are presented in Table 6. The inhibition zones of the UDES-NL-blended sunscreen showed activity against *Staphylococcus aureus* of 12 mm and 10 mm for 5% and 10% UDES-NL, respectively, and 0 mm (no effect) for −ve control (sunscreen alone) and 14 mm for +ve control (ciprofloxacin). Similarly, the zones of inhibition against *E. coli* were 0 mm (no effect), 10 mm, 6 mm and 10 mm for −ve control (sunscreen alone), +ve control, 5% UDES-NL, and 10% UDES-NL, respectively. The obtained result for *Staphylococcus aureus* showed that for 5% UDES-NL, the inhibition was better than the inhibition obtained for 10% UDES-NL, as compared with that for +ve control (ciprofloxacin). As compared to +ve control (ciprofloxacin), the obtained result for *Staphylococcus aureus* showed that the inhibition at 5% UDES-NL was better than the inhibition at 10% UDES-NL. Whereas, for *E. coli* the inhibition at 10% UDES-NL was better than the inhibition at 5% UDES-NL. In contrast, sunscreen alone showed an insignificant inhibition zone or no effect for both bacteria, *E. coli* and *Staphylococcus aureus* (*s*. *aureus*). Thus, the investigated antimicrobial effect was due to UDES-NL incorporation. Therefore, the prepared UDES-NL-blended sunscreen (BSLBS) not only shields the human skin from UV light but also acts as an anti-germ. Related results were presented by Madgundi *et al.* (2025),^24^ who studied the antimicrobial effects of nano-lignin obtained from rice straw lignin *via* acid hydrolysis for possible UV barrier applications in textile and sunscreen formulation.

**Table 6:** Values of the inhibition zone (mm) of CSS blended with 5% and 10% of UDES-NL; ciprofloxacin is taken as the +ve control and sunscreen is taken as the −ve control^a^

Microorganisms	Inhibition zone			
	−ve	+ve	5%	10%
*E. coli*	—	10 mm	6 mm	10 mm
*S*. *aureus*	—	14 mm	12 mm	10 mm

a −ve (negative control, CSS alone), +ve (positive control, ciprofloxacin).

## 4 Conclusion

In this study, the potential of nano-lignin (UDES-NL) as an active sunscreen filter for a wide-spectrum sunscreen was investigated by analyzing the UV transmittance and SPF values of the prepared nano-lignin-blended sunscreen. The UV transmittance analysis of the UDES-NL-blended sunscreen showed low transmittance in the spectrum range from 290 to 400 nm and 400 to 290 nm, forming a smooth broad-spectrum in between, with a pronounced effect for 10% UDES-NL. Similarly, the SPF analysis showed a significant change in the SPF value from SPF27 CSS to SPF30.89, SPF36.75, and SPF48.59 for 1% UDES-NL, 5% UDES-NL, and 10% UDES-NL, respectively. The rheological and antimicrobial analyses showed favorable properties for skin application, *i.e.*, easily spread when applied to skin, and antimicrobial activities, respectively. Moreover, the optical property of the prepared sunscreen was characterized, and the obtained result revealed that the prepared sunscreen could be utilized for different skin tones. Overall, based on the experimentally investigated results, SPF and UV transmittance, nano-lignin would be the future organic broad-spectrum sunscreen filter that helps to mitigate the impact of broad-spectrum UV radiation.

## Conflicts of interest

There are no conflicts to declare.

## Data Availability

The datasets generated during and/or analyzed in this work will be available from the corresponding author upon reasonable request.
